# The Space Omics and Medical Atlas (SOMA): new resource for medical research in deep space

**DOI:** 10.1002/mco2.780

**Published:** 2024-10-13

**Authors:** Hanwen Zhang, Yingxian Li, Guohui Zhong

**Affiliations:** ^1^ National Key Laboratory of Space Medicine China Astronaut Research and Training Center Beijing China; ^2^ School of Life Science Beijing Institute of Technology Beijing China

1

Recently, the Space Omics and Medical Atlas (SOMA) was presented by Overbey et al.,^1^ in *Nature*, showcasing the samples and physiological profiles of four crew members from SpaceX's Inspiration4 (I4) mission in 2021. Meanwhile, a series of SOMA‐related articles from more than 100 research groups in 25 countries have been published in *Nature* and its subjournals. I4 marked the first all‐civilians manned space mission globally, featuring a crew composed of a billionaire, a survivor of bone cancer, an air force veteran, and an Earth scientist. The SOMA (https://soma.weill.cornell.edu) is the first biobank of space medicine, expanding the human space omics data 10‐fold. It reveals the crew's physiological changes through a range of assays, including intelligent device monitoring, behavioral tests, and multiomics analysis, at various levels from molecules to cells and organs. This provides a reference for health monitoring, prevention, and clinical treatment, applicable to both deep space exploration and survival on Earth.

The field of manned spaceflight saw significant growth in recent years, yet the global space medicine remains nascent. The mental health of astronauts and civilian crew members can be affected by stress, noise, and confined space. The physiological well‐being of crew members can be impacted by unique factors such as microgravity and radiation exposure, which can induce the dysfunction of the cardiovascular system, musculoskeletal system, and immune system, even posing the life‐threatening risks during spaceflight or long‐term space habitation. It is imperative to elucidate the mechanisms of injury and identify biomarkers associated with spaceflight. This will enable us to assess the physiological statuses and develop the precision medicine for space, addressing the challenges of the “Second Space Age.”^2^

Four civilian astronauts (three men and one woman with the age of 29, 38, 42, and 51 years) completed the I4 orbital mission at 590.6 km elevation for 3 days. To explore the physiological variations profiles of I4 crew, 13 kinds of biospecimen (including whole blood, serum, peripheral blood mononuclear cells [PBMCs], dried blood spots, skin biopsies, stool, etc.) were collected across 10 time points (including three pre‐flight, three in‐flight, one immediately post‐flight, and three recovery time points) and the portable imaging devices, wearables, and multiomics analysis methods were conducted.^3^ The collection and analysis of biological samples are summarized (Figure 1) and some research highlights will be elaborated below.

**FIGURE 1:**
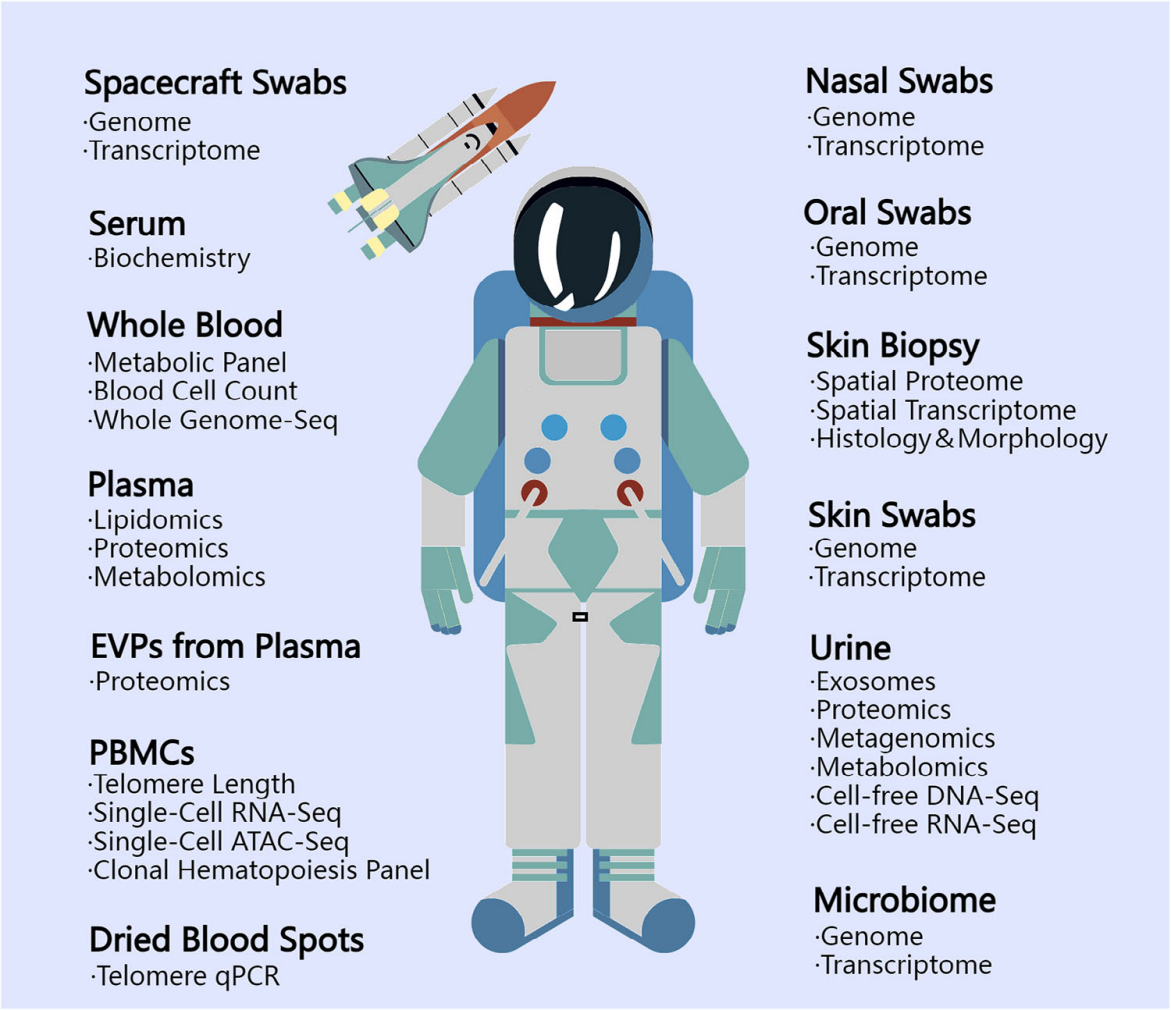
The collection and analysis of sample types in SOMA. Based on the multiomics research design, the whole blood, serum, peripheral blood mononuclear cells (PBMCs), plasma, extracellular vesicles, and particles (EVPs) derived from plasma, dried blood spots, oral swabs, nasal swabs, skin biopsies, skin swabs, capsule swabs, urine, and stool were collected and banked.

The novel RNA fingerprints of spaceflight were included in SOMA. Overbey et al.^1^ found 95 regions of interest across outer epidermis, inner epidermis, outer dermis, and vasculature on skin biopsies by the spatially resolved transcriptomics. Especially, the melanocyte abundance decreased significantly in the inner epidermis and outer dermis during post‐flight. Distinctly, the cell‐free RNA‐sequencing profiling displayed an obvious distinction during pre‐flight, in‐flight, and post‐flight. The cell type was inferred to exhibit that the abundance of hepatocytes, kidney endothelial cells, hematopoietic stem cells, and melanocytes increased after spaceflight. Additionally, the direct RNA‐sequencing and deep RNA‐seq were used to show differentially expressed genes (DEGs), differential methylation, and the evidence of radiation and telomere shortening response.

The human microbiome was emphasized in SOMA. Tierney et al.^4^ used shotgun metagenomics and metatranscriptomics alongside single‐nuclei immune cell profiling to examine the crew's microbiome of 10 body sites. They observed that most disturbance of sites were transient during flight except for the oral microbiome. For example, the abundance of *Fusobacteriota* related with immune cell gene expression persistently increased, showing a risk of tooth diseases such as gingivitis. The skin microbiome was altered by the environment and co‐habitants, but the beta‐diversity had no change. Besides, researchers observed significant increases in phage proteins, toxin–antitoxin systems, and antibiotic‐related/heavy metal pathways during flight.

The in‐depth immune system map was drawn in SOMA. Kim et al.^5^ found that levels of the proinflammatory factors such as tumor necrosis factor‐α and C‐reaction protein, and the chemokines such as Interferon‐γ‐inducible protein 10, epithelial cell‐derived neutrophil‐activating peptide 78, and fractalkine increased during spaceflight via a complete blood count and a comprehensive metabolic panel method. The single‐nuclei RNA sequencing of PBMCs was used to demonstrate the gene regulatory shift. There were 905 DEGs, most DEGs were reversed after spaceflight. The downregulated DEGs were enriched in ribosomal translation, mitochondrial metabolism, UV response, TCF21 targets, and immune function pathways. The upregulated DEGs were enriched in response to stimulus and signaling. By contrast, there were 8564 DEGs in the National Aeronautics and Space Administration (NASA) Twins Study due to one‐year spaceflight. Overall, short‐term flights resulted in more modest changes than long‐term flights and most of them were stress related. To explore the gene regulatory variation and chromatin accessibility in different cell types, researchers clarified that the CD14 monocytes responded to spaceflight were the most obvious, CD4 T and CD8 T cells were the least by performing the single‐nuclei ATAC‐seq. Additionally, the correlation of upregulated gene expression with more accessibility at the transcription start sites was also illustrated.

In summary, the SOMA database covers the 2911 biological samples and hundreds of terabytes of data. It also integrates physiological data from astronauts on previous missions, including the NASA Twins Study, Japan Aerospace Exploration Agency Cell‐free Epigenome Study, Axiom, and Polaris. The crew of I4 mission was comprised of the individuals of varying ages and genders. However, to meet the demands of long‐term and high‐altitude spaceflight missions, the study cohort needs further expansion and the spaceflight time needs expansion. Although nearly 50 articles based on SOMA have been published, the efficient utilization of this database is yet to be further explored and developed. Artificial intelligence analysis based on machine learning may empower space medicine research based on big data and serve as a powerful tool for real‐time physiological monitoring and predictive protection measures, with the potential to assess individual risks and tailor countermeasures for crew members. Inspiringly, there is no doubt that the creation of SOMA has opened the second era of human space exploration and will provide research references and guidance for the precision medicine in space medicine and the ground human health.

## AUTHOR CONTRIBUTIONS

Hanwen Zhang drafted the manuscript. Yingxian Li provided valuable suggestions and ideas for revisions. Guohui Zhong designed the structure of the manuscript and provided revisions and guidance.

## CONFLICT OF INTEREST STATEMENT

The authors declare no conflict of interest.

## ETHICS STATEMENT

Not applicable.

## Data Availability

Not applicable.
